# Investigation of heavy metals in frequently utilized medicinal plants collected from environmentally diverse locations of north western India

**DOI:** 10.1186/2193-1801-2-676

**Published:** 2013-12-17

**Authors:** Alpana Kulhari, Arun Sheorayan, Somvir Bajar, Susheel Sarkar, Ashok Chaudhury, Rajwant K Kalia

**Affiliations:** Department of Bio and Nanotechnology, Guru Jambheshwar University of Science and Technology, Hisar, 125001 India; Department of Environmental Science and Engineering, Guru Jambheshwar University of Science and Technology, Hisar, 125001 India; Indian Agricultural Statistics Research Institute, Library Avenue, Pusa New Delhi, India; Central Arid Zone Research Institute (CAZRI), Jodhpur, Rajasthan 342003 India

**Keywords:** AAS, Heavy metals, Herbal plants, Soil pollution, Toxicity

## Abstract

The increasing prevalence of environmental pollution, especially soil contamination with heavy metals has led to their uptake in the human food chains through plant parts. Accumulation and magnification of heavy metals in human tissues through consumption of herbal remedies can cause hazardous impacts on health. Therefore, chemical profiling of nine heavy metals (Mn, Cr, Pb, Fe, Cd, Co, Zn, Ni and Hg) was undertaken in stem and leaf samples of ten medicinal plants (*Acacia nilotica*, *Bacopa monnieri*, *Commiphora wightii*, *Ficus religiosa*, *Glycyrrhiza glabra*, *Hemidesmus indicus*, *Salvadora oleoides*, *Terminalia bellirica*, *Terminalia chebula* and *Withania somnifera*) collected from environmentally diverse regions of Haryana and Rajasthan states in North-Western India. Concentration of all heavy metals, except Cr, was within permissible limits in the tested stem and leaf samples. Leaf samples had consistently more Cr compared to respective stem samples with highest concentration in leaf samples of *Bacopa monnieri* (13.19 ± 0.0480 ppm) and stem samples of *Withania somnifera* (4.93 ± 0.0185 ppm) both collected from Bahadurgarh (heavy industrial area), Haryana. This amount was beyond the permissible limit of 2.0 ppm defined by WHO for raw herbal material. Other two most perilous metals Pb (2.64 ± 0.0260) and Cd (0.04 ± 0.0274) were also recorded in Bahadurgarh region, although below permissible limits. Concentration of Hg remained below detectable levels in all the leaf and stem samples tested. These results suggested that cultivation of medicinal plants and other dietary herbs should be curtailed near environmentally polluted especially industrial areas for avoidance of health hazards.

## Introduction

Herbal medicines being natural are preferred over synthetic remedies by a major section of the world. Since times immemorial, ancient Indians, Egyptians, Chinese and others have employed a variety of plants and plant products for curing all kinds of ailments. Approximately, 25000 plant based formulations are available in the indigenous medical texts (Gupta et al., 2004). Also, the modern pharmacopoeia contains at least 25% drugs derived from plants and many others which are synthetic analogues built on prototype compounds isolated from plants (Rao et al., 2004). The World Health Organization (WHO) estimated that 80% of the population of developing countries relies on traditional medicines, mostly plant drugs, for their primary health care needs (Planning Commission Report, 2000). Complementary and alternative medicine has received global attention in the last few years with a frequency of 31.4% population in industrialized societies, 42–69% in United States, 71% Canadians and 90% British population consuming dietary supplements or natural health products (vitamins, minerals, amino acids, essential fatty acids, herbal products, traditional Chinese medicines, homeopathic medicines, and probiotics) for treatment, including elimination of disease causing agents, avoidance of side effects and for getting quality life (Szeto, 2007). The world market for herbal medicine, including herbal products and raw materials has been estimated to have an annual growth rate between 5 and 15%. Total global herbal drug market is estimated at US $62 billion and is expected to grow to US $5 trillion by the year 2050 (Joshi et al., 2004).

Accumulation of toxic industrial effluents in the soil is continuously increasing due to fast urbanization and extensive pollution of the environment. Among these toxic substances, presence of heavy metals (HMs; atomic weights 63.5–200.6 g mol^−1^ and a specific gravity greater than 5 g cm^−3^) which are ubiquitous in nature, cause serious harmful effects on living organisms (Nies, 1999; Lee and Lee, 2002). Plants are sensitive to environmental conditions and they accumulate these HMs in their harvestable parts (via root uptake, foliar adsorption and deposition of specific elements in leaves) and intensity of this uptake process changes the overall elemental composition of the plant (Olajire and Ayodele, 2003). Uptake, accumulation and concentration of HMs in plants is influenced by various attributes including atmospheric depositions (depend on traffic densities, metal mining and smelting operations), concentration and bioavailability of HMs in soil (through addition of pesticides and sewage sludge), the nature of soil where herbs are grown (pH and organic matter concentration), individual plant performance (degree of maturity of the plant, time of harvest) and manufacturing conditions of herbal drugs (grinding weights, lead-releasing containers and manufacturing utensils) etc (Nwoko and Mgbeahuruike, 2011). Different HMs have different transmitting rates from soil-to-plant, based on transfer coefficients of metals viz: Cd, Tl and Zn are readily taken up by plants because of higher transfer coefficient, whereas Cu, Co, Cr, and Pb are stably bound to the soil structures and show minimum transfer to plants from soil due to lower transfer coefficient (Kloke et al., 1984). Some metals (Mg, Mn and Zn) play a vital role in proper growth and development of the plant being directly or indirectly involved in various biological functions of enzyme activation and molecular metabolism. Very little information is available about potential influence of metals on pharmacological activity of natural drugs obtained from medicinal plants. Metal mediated hazardous impacts can be direct or indirect via binding of metals with pharmacologically active substances or by manipulating the pharmacokinetics (Weber and Konieczyński, 2003).

Consumption of raw herbal drugs from the medicinal plants, grown in polluted sites can cause serious consequences on human health. Higher levels of these elements are carcinogenic and affects the central nervous system (Hg, Pb and As), cause kidney damage and liver dysfunction (Hg, Pb, Cd and Cu), are toxic to skin, bones and teeth (Ni, Cd, Cu and Cr) and have adverse effects on memory and reproductive system (Hussain et al., 2011). For getting desirable therapeutic benefits, quality of these herbal products must be ensured in terms of metal contamination. Therefore, there is an urgent need for quick assessment of these heavy metals in medicinal plants to control the level of contaminants in herbal raw materials. Generally, three methods-atomic absorption spectrophotometry (AAS), inductively coupled plasma (ICP) and neutron activation analysis (NAA) (Kunle et al., 2012) have been employed for quantitative estimation of metals present in herbal raw material as an admixture or in trace amounts.

This study was designed to investigate the levels of nine heavy metals (Mn, Cr, Pb, Fe, Cd, Co, Zn, Ni and Hg) in leaf and stem samples of ten medicinal plants (*Acacia nilotica*, *Bacopa monnieri*, *Commiphora wightii*, *Ficus religiosa*, *Glycyrrhiza glabra*, *Hemidesmus indicus*, *Salvadora oleoides*, *Terminalia bellirica*, *Terminalia chebula* and *Withania somnifera*) collected from environmentally diverse regions of North Western India (Haryana and Rajasthan states) using AAS. The collection sites were shortlisted on the basis of presence of contaminated soil and atmospheric polluting units (presence of industries, heavy traffic sites and site located near to petrol pumps) whereas the medicinal plants were selected on the basis of their significant importance in the Ayurveda along with their frequent utilization in novel pharmaceutical preparations, preparation of traditional drug formulations, food supplements and their diverse medicinal properties. General description and economic importance of medicinal plants undertaken in the present study is given in Table 1.

**Table 1:** **General description and economic importance of medicinal plants used in the study**

Scientific name	Common name	Family	Habit	Part used	Medicinal properties	References
*Acacia nilotica* (L.) Willd. Ex Delile	Babul, kikar	Mimosaceae	Medium sized tree	Bark, roots, seeds, gum, pods	Used to cure diarrhea, Aphrodisiac, dressing of ulcers, Alzheimer’s diseases, wound ulcers, leprosy, leucoderma, small pox, skin diseases, biliousness, burning sensation and toothache.	Ali et al. (2012)
*Bacopa monnieri* (L.) Pennell	Brahmi	Plantaginaceae	Perennial herb	Leaves	Improve memory capacity, intellectual activity and enhance the immune function by increasing immunoglobulin production.	Morgan and Stevens (2010) Yamada et al. (2011)
*Commiphora wightii* (Arn) Bhandari	Guggul	Burseraceae	Balsamiferous woody shrub	Bark, stem	Active ingredients E and Z guggulsterone in oleoresin gum have lipid and cholesterol lowering activities along with acting as anti- cancerous compound.	Kulhari et al. (2012)
*Ficus religiosa* L.	Peepal	Moraceae	Large deciduous tree	Bark, leaves, seeds, fruits,	Used to cure asthma, cough, sexual disorders, gonorrhea, skin diseases, scabies, hiccup, tuberculosis, fever and paralysis.	Makhija et al. (2010)
*Glycyrrhiza glabra* L.	Mulethi	Fabaceae	Perennial herb	Roots	Popularly used to treat ileitis, lung inflammation, peptic ulcers, leaky gut syndrome and irritable bowel syndrome.	Krausse et al. (2004)
*Hemidesmus indicus* L.R.Br.	Anantmul	Apocynaceae	Perennial climber	Leaves, stem	Used in inflammation, cuts, wounds, burns, skin and blood diseases, ulcers and immunological disorders.	Saravanan and Nalini (2007)
*Salvadora oleoides* Decne.	Jaal, Pilu	Salvadoraceae	Small tree	Leaves, roots	The leaves have anti-inflammatory, analgesic and antiulcer activities.	Yadav et al. (2008)
*Terminalia bellirica* (Gaertn.) Roxb.	Bahera	Combretaceae	Long tree	Leaves, bark, seed, roots, flower	Used in the treatment of gastric ulcer, constipation, general debility and piles.	Motamarri et al. (2012)
*Terminalia chebula* Retz.	Harītak, Harad	Combretaceae	Medium to large deciduous tree	Leaves, bark, fruits	Used to cure anemia, anorexia, leprosy, diarrhoea, bleeding piles, gout, arthritis, epilepsy abdominal pain and asthma.	Rao and Srinivas (2006)
*Withania somnifera* (L.) Dunal	Ashwagandha	Solanaceae	Erect branched shrub	Leaves, stem, roots, seed	Used to promote health and longevity along with revitalizing of body in debilitated conditions and increasing the capability of the individual to resist adverse environmental factors.	Weiner and Weiner (1994)

## Materials and methods

Leaf and stem samples of *Acacia nilotica*, *Bacopa monnieri*, *Commiphora wightii*, *Ficus religiosa*, *Glycyrrhiza glabra*, *Hemidesmus indicus*, *Salvadora oleoides*, *Terminalia bellirica*, *Terminalia chebula* and *Withania somnifera* were collected separately from different geographical locations of Haryana and Rajasthan states of India. Sampling was done from five districts namely Jhunjhunu, Churu, Bahadurgarh, Fathebad and Mahendergarh (Table 2). The collected material was washed thoroughly with running tap water followed by washing with deionized autoclaved water to remove the dust particles and possible parasites. They were shade dried, powdered and stored in closed air tight bottles for further experimentation.

**Table 2:** **Collection of medicinal plants from Rajasthan and Haryana states**

Plant name	State	District	Latitude	Longitude	Status
*Acacia nilotica*	Rajasthan	Jhunjhunu	28.13° N	75.40° E	Wild
*Bacopa monnieri*	Haryana	Bahadurgarh	28.68° N	76.92° E	Wild
*Commiphora wightii*	Haryana	Mahendergarh	28.27° N	76.15° E	Wild
*Ficus religiosa*	Rajasthan	Jhunjhunu	28.13° N	75.40° E	Wild
*Glycyrrhiza glabra*	Haryana	Bahadurgarh	28.68° N	76.92° E	Wild
*Hemidesmus indicus*	Haryana	Fathebad	29.52° N	75.45° E	Wild
*Salvadora oleoides*	Rajasthan	Churu	26.60° N	75.45° E	Wild
*Terminalia bellirica*	Haryana	Fathebad	29.52° N	75.45° E	Wild
*Terminalia chebula*	Haryana	Fathebad	29.52° N	75.45° E	Wild
*Withania somnifera*	Haryana	Bahadurgarh	28.68° N	76.92° E	Wild

Heavy metal analysis was done according to AOAC (1995) using flame atomic absorption spectroscopy in ten medicinal plants using wet digestion method (Meena et al., 2010). Standards of Fe, Pb, Mn, Cr, Zn, Cd, Co, Hg and Ni procured from Merck, Germany, were used as reference analytes for quantitative estimation of heavy metals as well as accurate calibration and quality assurance of each analyte. The standard stock solutions (1000 ppm) were diluted to obtain working standard solutions ranging from 1 ppm to 10 ppm and stored at 4°C. An acidity of 0.1% nitric acid was maintained in all the solutions. A calibration curve was plotted between measured absorbance and concentration (ppm). All the samples were analyzed in triplicate using Flame Atomic Absorption Spectrophotometer (AA 6300 Shimadzu, Japan) with Shimadzu, Wizard software. Optimized operating parameters for FAAS (Flame Atomic Absorption Spectrophotometer) of various heavy metals are listed in Table 3.

**Table 3:** **Operating parameters for FAAS of heavy metals**

Parameters	Heavy metals								
	Mn	Cr	Pb	Fe	Cd	Ni	Co	Zn	Hg
Wavelength (nm)	279.5	357.9	283.3	248.3	228.8	232.0	240.7	213.9	253.7
Slit (nm)	0.7	0.7	0.7	0.7	0.7	0.7	0.7	0.7	0.7
Acetylene flow rate (l/min)	1.8	1.8	1.8	1.8	1.8	1.8	1.8	1.8	1.8
HCL current (mA)	6	6	6	6	6	6	6	6	6
Air flow rate ( l/min)	15	15	15	15	15	15	15	15	15

For heavy metal assessment, 5 g powder of each plant sample was taken into separate crucibles for charring for 3–4 hours. After charring, the samples were placed in the Muffle furnace at 500°C for 6 hrs for ashing and were then cooled in the desiccators. Samples were digested in 20 ml mixture of concentrated acids (nitric and perchloric acid in 9:1 ratio) for 3 hours in a water bath maintained at 70°C for dissolving the contents until a clear brownish solution was obtained using wet digestion method. After cooling, these solutions were reconstituted to 20 ml volume with deionized autoclaved water. Each sample was filtered using whatmann filter paper (pore size 0.45 μ, Axiva) and stored in closed acid-washed glass vials. The stored samples were further used for analysis of heavy metals using flame atomic absorption spectroscopy. All experiments were done in triplicate for precision and accuracy of the results. Concentration of each metal was determined from absorbance value of each replicate and articulated in ppm on a dry-weight basis of the plant sample. Finally data was subjected to standard error calculation using SAS 9.3

## Results and discussion

**Figure 1:**
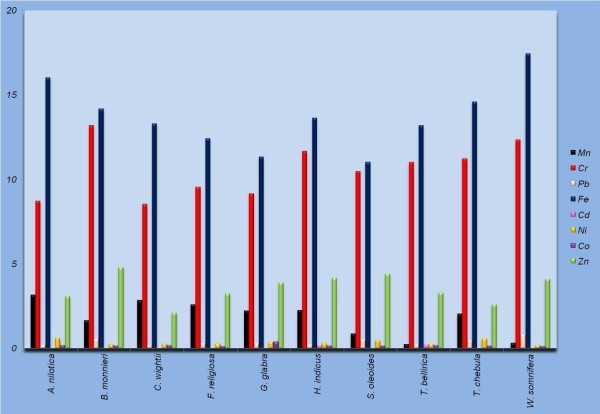
**Heavy metal concentration in leaf samples of ten medicinal plants collected from different geographical regions.**

**Figure 2:**
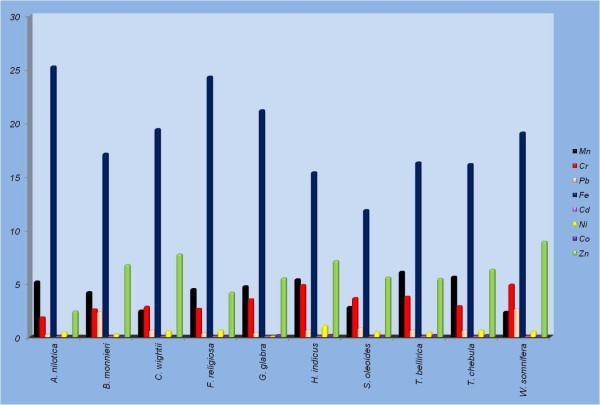
**Heavy metal concentration in stem samples of ten medicinal plants collected from different geographical regions.**

Atmosphere and soil are continuously being polluted with chemicals and heavy metals due to dynamic development of industries and motorization along with extensive use of pesticides and fertilizers. In turn, these pollutants and heavy metals are getting deposited in the plants growing in the polluted areas, which subsequently enter the human food chain via plant parts and/or extracts. Results of analysis of nine heavy metals (Mn, Cr, Pb, Fe, Cd, Co, Zn, Hg and Ni) done in stem and leaf samples of ten medicinal plant species are summarized in Tables 4 and 5 respectively. Significant difference in heavy metal concentration was observed among the plants in the two tissue samples (leaf and stem) collected from diverse geographical locations (Figures 1 and 2).

**Table 4:** **Concentration of heavy metals in stem samples of medicinal plants**

		Mean heavy metal concentration in stem samples (ppm)								
S/N	Plant species	Mn	Cr	Pb	Fe	Cd	Ni	Co	Zn	Hg
1	*Acacia nilotica*	5.22 ± 0.1040	1.87 ± 0.0202	0.25 ± 0.0088	25.30 ± 0.1464	BDL	0.45 ± 0.0240	0.04 ± 0.0088	2.42 ± 0.2173	BDL
2	*Bacopa monnieri*	4.23 ± 0.0744	2.62 ± 0.0240*	2.34 ± 0.0173	17.16 ± 0.1763	0.02 ± 0.0033	0.26 ± 0.0284	0.05 ± 0.0033	6.75 ± 0.1223	BDL
3	*Commiphora wightii*	2.51 ± 0.0176	2.87 ± 0.0202*	0.63 ± 0.0088	19.45 ± 0.2334	BDL	0.55 ± 0.0173	0.02 ± 0.0088	7.74 ± 0.0802	BDL
4	*Ficus religiosa*	4.52 ± 0.0176	2.67 ± 0.0371*	0.36 ± 0.0317	24.35 ± 0.1808	0.02 ± 0.0010	0.64 ± 0.0145	0.12 ± 0.01201	4.17 ± 0.0736	BDL
5	*Glycyrrhiza glabra*	4.78 ± 0.0240	3.56 ± 0.0305*	0.41 ± 0.0185	21.21 ± 0.0173	0.03 ± 0.0120	0.06 ± 0.0120	0.20 ± 0.0185	5.52 ± 0.0202	BDL
6	*Hemidesmus indicus*	5.43 ± 0.0173	4.89 ± 0.0202*	0.66 ± 0.0352	15.42 ± 0.0176	0.03 ± 0.0145	1.07 ± 0.0057	0.23 ± 0.0881	7.13 ± 0.0115	BDL
7	*Salvadora oleoides*	2.85 ± 0.0218	3.68 ± 0.0202*	0.83 ± 0.0088	11.89 ± 0.0317	BDL	0.47 ± 0.0115	0.13 ± 0.0173	5.59 ± 0.0317	BDL
8	*Terminalia bellirica*	6.13 ± 0.0176	3.80 ± 0.0450*	0.63 ± 0.0115	16.34 ± 0.0392	0.03 ± 0.0088	0.43 ± 0.0173	0.21 ± 0.0290	5.46 ± 0.0545	BDL
9	*Terminalia chebula*	5.67 ± 0.0384	2.92 ± 0.0202*	0.62 ± 0.0384	16.19 ± 0.0633	0.03 ± 0.0152	0.64 ± 0.0750	0.20 ± 0.0589	6.32 ± 0.0202	BDL
10	*Withania somnifera*	2.39 ± 0.0218	4.93 ± 0.0185*	2.64 ± 0.0260	19.13 ± 0.0176	0.04 ± 0.0088	0.53 ± 0.0296	0.14 ± 0.0152	8.93 ± 0.0264	BDL

*Beyond permissible limit defined by WHO.

**Table 5:** **Concentration of heavy metals in leaf samples of medicinal plants**

		Mean heavy metal concentration in leaf samples (ppm)								
S/N	Plant species	Mn	Cr	Pb	Fe	Cd	Ni	Co	Zn	Hg
1	*Acacia nilotica*	3.16 ± 0.0371	8.73 ± 0.0260*	0.21 ± 0.0208	16.01 ± 0.0642	BDL	0.59 ± 0.0202	0.19 ± 0.0284	3.09 ± 0.0305	BDL
2	*Bacopa monnieri*	1.67 ± 0.0425	13.19 ± 0.0480*	0.54 ± 0.0417	14.19 ± 0.0633	0.03 ± 0.0088	0.23 ± 0.0317	0.15 ± 0.0115	4.80 ± 0.0907	BDL
3	*Commiphora wightii*	2.84 ± 0.0688	8.53 ± 0.0317*	0.21 ± 0.0145	13.30 ± 0.0264	BDL	0.26 ± 0.0348	0.20 ± 0.0200	2.10 ± 0.0173	BDL
4	*Ficus religiosa*	2.60 ± 0.0392	9.54 ± 0.0202*	0.32 ± 0.0240	12.42 ± 0.0272	0.03 ± 0.0057	0.30 ± 0.0296	0.14 ± 0.0120	3.25 ± 0.0240	BDL
5	*Glycyrrhiza glabra*	2.23 ± 0.0305	9.17 ± 0.0173*	0.32 ± 0.0233	11.33 ± 0.0296	0.03 ± 0.01660	0.38 ± 0.0233	0.40 ± 0.0251	3.89 ± 0.0348	BDL
6	*Hemidesmus indicus*	2.26 ± 0.0260	11.68 ± 0.0480*	0.25 ± 0.0088	13.64 ± 0.0115	0.16 ± 0.0260	0.32 ± 0.0173	0.16 ± 0.0251	4.18 ± 0.0212	BDL
7	*Salvadora oleoides*	0.89 ± 0.0458	10.48 ± 0.0440*	0.52 ± 0.0202	11.03 ± 0.0497	BDL	0.48 ± 0.0193	0.15 ± 0.0208	4.40 ± 0.0240	BDL
8	*Terminalia bellirica*	2.60 ± 0.0348	11.02 ± 0.0808*	0.25 ± 0.0120	13.21 ± 0.0202	0.21 ± 0.0176	0.26 ± 0.0257	0.18 ± 0.0145	3.28 ± 0.0145	BDL
9	*Terminalia chebula*	2.04 ± 0.0264	11.23 ± 0.0887*	0.48 ± 0.0290	14.59 ± 0.0352	0.02 ± 0.0120	0.57 ± 0.0264	0.17 ± 0.0264	2.58 ± 0.0237	BDL
10	*Withania somnifera*	0.34 ± 0.0152	12.34 ± 0.0458*	0.81 ± 0.0360	17.44 ± 0.0202	0.04 ± 0.0274	0.19 ± 0.0371	0.14 ± 0.0135	4.10 ± 0.0360	BDL

*Beyond permissible limit defined by WHO.

**Figure 3:**
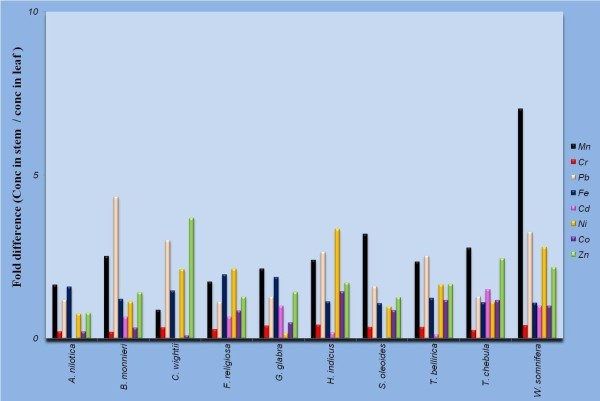
**Fold difference in concentration of heavy metals in stem samples compared to respective leaf samples (conc. in stem sample/conc. in leaf sample).**

Manganese (Mn) is known as an essential trace element which acts as cofactor for many enzymes. It is less toxic than any other metal, however, can cause neurological disorders if its concentration exceeds 5 mg/m^3^ due to continuous exposure to manganese dust and fumes. In the present study, a wide variation occurred in Mn concentration in stem and leaf samples, with highest content in the stem samples of *T. bellirica* (6.13 ppm) and leaf samples of *A. nilotica* (3.16 ppm) collected from Fathebad and Jhunjhunu, respectively. Mn concentration was more in the stem samples, ranging from 1.65 fold in *A. nilotica* to 7.03 fold in *W. somnifera*, compared to respective leaf samples except *C. wightii* wherein leaf samples accumulated more Mn (Figure 3). Chromium (Cr), regarded as one of the most toxic pollutants in the world is released by tanneries, steel industries, and sewage sludge applications along with alloys in motor vehicles. Its concentrations between 5–30 mg/kg are considered critical for plants as it causes heavy reduction in plant growth and yield. The permissible limit for Cr in raw herbal materials is 2.0 ppm and that for finished products is 0.02 mg/day (WHO, 2007). All the samples tested, except *A. nilotica* stem had Cr concentrations beyond the permissible limits defined by WHO. Cr concentration varied between 1.87 ± 0.0202 (A. *nilotica*) to 4.93 ± 0.0185 ppm (*W. somnifera*) in the stem samples while it varied from 8.53 ± 0.0317 (*C. wightii*) to 13.19 ± 0.0480 ppm (*B. monnieri*) in leaf samples. Cr concentration was 2.39 (*H. indicus*) to 5.03 (*B. monnieri*) fold more in leaf samples compared to respective stem samples. Lead (Pb) is highly hazardous for plants, animals and microorganisms. Continuous consumption of fertilizers, fuel combustion and sewage sludge are the major reasons leading to escalation in Pb pollution. The plant tissues tested (stem and leaf) contained extremely lower concentrations of Pb compared to the permissible limit of 10 ppm defined by WHO (2007). Maximum amount of Pb was found in stem (2.64 ± 0.0260 ppm) and leaf (0.81 ± 0.0360 ppm) samples of *W. somnifera* collected from Bahadurgarh. Pb concentration was comparable in stem and leaf samples of *A. nilotica*, *F. religiosa*, *G. glabra* and *T. chebula*, however, it was 3.0–.4.5 fold more in stem samples of *C. wightii*, *W. somnifera* and *B. monnieri*.

Iron (Fe) is an important trace element and iron-protein mixtures play a vital role in metabolism in all living organisms. However, Fe overdose has been one of the leading reasons of death caused by toxicological agents in children younger than 6 years of age. Individuals demonstrate signs of gastro-intestinal toxicity after ingestion of more than 20 mg/kg iron while moderate intoxication occurs when ingestion of elemental Fe exceeds 40 mg/kg and ingestions exceeding 60 mg/kg can cause severe toxicity and may be lethal also (Spanierman, 2011). Here, highest content of Fe was found in *A. nilotica* stem samples (25.30 ± 0.1464 ppm) and *W. somnifera* leaf samples (17.44 ± 0.0202 ppm) collected from Jhunjhunu and Bahadurgarh, respectively. However, all concentrations were within the permissible limits. On the other hand, Annan et al., (2010) had reported essential element mediated toxicity due to higher accumulation of Fe (beyond the acceptable range 1000 μg/day) in *Ocimum canum*, *Clausena anisata* and *Rauwolfia vomitoria* collected from Ghana using wet digestion AAS. Fe concentrations were comparable (1.1–1.2 fold in *S. oleoides*, *W. somnifera*, *T. bellirica*, *T. chebula*, *H. indicus*, *B. monnieri* or higher (1.5–2.0 fold in *A. nilotica*, *C. wightii*, *F. religiosa*, *G. glabra*) in stem samples compared to respective leaf samples.

Cadmium (Cd) is another hazardous heavy metal which can cause significant reduction in plant yield at concentrations ranging from 5–30 mg/kg. Recently, it is gaining more attention due to wide occurrence in water, soil, milk, dietary products, medicinal plants and herbal products. The major sources leading to accumulation of cadmium in soil and plants are phosphate fertilizers, non-ferrous smelters, lead and zinc mines, sewage sludge application and combustion of fossil fuels. All the samples analyzed in this study had Cd concentration within the acceptable range of 0.3 ppm recommended by WHO (2007) for raw herbal material. Maximum amount of Cd was found in *W. somnifera* (0.04 ppm in both stem and leaf samples) collected from Bahadurgarh region, whereas, it remained undetected in stem samples of *A. nilotica* (Jhunjhunu), *C. wightii* (Mahendergarh) and *S. oleoides* (Churu). Cd concentration was comparable in stem and leaf samples of other plants except *T. bellirica* and *H. indicus* wherein Cd concentration was 5–7 fold more in leaf samples. Nickel (Ni) and Cobalt (Co) are trace elements required for a variety of biological processes. Ni is directly coordinated by proteins, whereas, Co is mainly used as a component of vitamin B_12_ (Zhang et al., 2009). Nickel was recognized as an allergen of the year in 2008 by the American Contact Dermatitis Society and its minimal risk level was set to 0.2 μg/m^3^ for inhalation during 15–364 days, however, no limit has been set for food stuffs (Bhat et al., 2010). In the present investigation, less than 2 ppm Ni concentration was found in all the samples. Ni concentration was 2.0–3.3 folds more in stem samples in *C. wightii*, *F. religiosa*, *H. indicus* and *W. somnifera*. Concentration of Co, another major cause of contact dermatitis next only to nickel and chromium (Basketter et al., 2003), was less than 0.2 ppm in all the stem and leaf samples. Co concentration was comparable in stem and leaf samples in most of the plants studied except *A. nilotica*, *B. monnieri*, *C. wightii* and *G. glabra* where it was more in leaf samples.

Zinc (Zn) is an essential component of thousands of proteins in plants, although it is toxic in excess quantities. Maximum amount of zinc was found in stem samples of *W. somnifera* (8.93 ± 0.0264 ppm), followed by *C. wightii* (7.74 ± 0.0802 ppm) while in leaf samples highest content was found in *B. monnieri* (4.80 ± 0.0907 ppm) followed by *H. indicus* (4.18 ± 0.0212 ppm). Concentration of Zn was 2.0–3.7 folds more in stem samples of *C. wightii*, *T. chebula* and *W. somnifera*, while it was comparable in stem and leaf samples of other species tested. In Canada, 0.2 ppm has been prescribed as the limit of mercury (Hg) in raw herbal material, whereas in finished herbal products it is 0.02 mg/day. In China and Singapore however, 0.5 ppm limit has been proposed by WHO (2007). Hg concentration was below detectable limits in all the leaf and stem samples analyzed during this study. Radwan and Salama (2006) evaluated the level of Pb, Cd, Cu and Zn in various fruits and vegetables sold in Egyptian markets and found that their concentration was significantly higher but below the acceptable range in strawberries, cucumber, date and spinach. On the other hand, level of four most hazardous heavy metals Pb, Cd, Cu and Cr were found beyond the permitted limits in *Glycyrrhiza glabra*, *Onosma bracteatum*, *Viola odorata*, *Foeniculum vulgare*, *Cuminum cyminum*, *Coriandrum sativum* and *Zingiber officinalis* collected from southern, eastern, and western zones of Karachi, Pakistan (Hina et al., 2011).

Significant variation in metal concentration depending upon the plant species was recorded in the present study. Concentration of all heavy metals apart from Cr was found below the permissible limits in the stem and leaf samples of the ten medicinal plant species tested irrespective of their collection sites. Leaf samples had consistently more Cr compared to respective stem samples. Similarly, ten medicinal plants studied by Ajasa et al., (2004) collected from different areas of Ogbomoso revealed variable metallic levels. A higher mean level was obtained regarding Pb, Zn and Cu in *Azadirachta indica* and *Hyptis suaveolens*. Gajalakshmi et al., (2012) analyzed six heavy metals (Ni, Cu, Cr, Zn, Mn, Pb) in four medicinal plant species and revealed similar results. They reported that Cu concentration was higher in leaf samples of all the species, although lower than permissible limit, whereas Cr concentration was at higher level in all the plants, Zn and Mn were below the tolerable edge, whereas Ni and Pb were completely missing in all the analyzed samples.

The rise in Cr concentration in Bahadurgarh region (heavy industrial area located at the edge of national capital New Delhi) in the present study can be attributed to fast industrialization, higher vehicle density and rapid urbanization. Princewill-Ogbonna and Ogbonna (2011) also reported that fast urbanization in developed cities is responsible for drastic increase of metal concentration in surrounding areas and plant species growing on those sites. They reported that higher liberation of vehicular pollutants in Aba city of Nigeria exhibited variable accumulation of heavy metals in different medicinal plants as demonstrated by AAS mediated chemical profiling. Similarly, analytical profile of Pb, Cd, Cr, Ni, Sn, Zn, Mn, Cu and Fe exhibited concentrations at levels higher than permissible limits in various spice and medicinal plant species collected from exportation areas of Egypt (Abou-Arab and Abou Donia, 2000). Baranowska et al., (2002) evaluated the level of five heavy metals (Pb, Zn, Cd, Ni and Mo) in six medicinal plants through FAAS from different heavily polluted locations and concluded that all the metals were higher in concentration at Express highways, Railway stations and urban roads.

The present study concluded that bio-accumulation of heavy metals increased with rise in their trophic levels, therefore, constant utilization of the herbs and vegetables may escort its metal level in humans as also reported by Akinola et al., (2008). Highly contaminated sites are responsible for heavy metal accumulation in plant parts, utilized either raw or as finished herbal products. According to an All India Ethnobiological Survey carried out by the Ministry of Environment & Forests, Government of India, there are over 8000 species of plants being used by the people of India for medicinal purposes (Planning Commission Report, 2000). Therefore, the raw material collection sites need to be considered for their sporadic assessment for quality declaration and utilization. The medicinal plants-related trade in India estimated at Rs 5000 crores per annum with an annual growth rate of 7–15% (Joshi et al., 2004), may face serious consequences if heavy metal accumulation beyond permissible limits is detected. Therefore, assessment of these hazardous metals in raw herbal material and finished products must be undertaken compulsorily. Recently, studies have been initiated for detection of heavy metals in many medicinal plants (Hussain et al., 2011; Baye and Hymete, 2010; Meena et al., 2010) utilizing AAS. Concentration of Pb and Cd was more than the permissible limits recommended by WHO for some medicinal plants analyzed by Baye and Hymete (2010). Instrumental neutron activation analysis and AAS of five heavy metals (Cr, Ni, Cd, As and Pb) and seven essential metals (V, Mn, Fe, Co, Cu, Zn, Se) from Khetri copper mine and those in fertile soils of Haridwar, India exhibited a wide variation in heavy metal concentration sketch. Concentrations of Cu, Cr, Cd and Pb were higher in Khetri copper mine as compared to Haridwar samples, whereas Ni, As and other essential metals showed little variation in both the collection sites (Maharia et al., 2010). It was also reported that *Withania somnifera* showed highest bio-accumulation of metals as compared to *Ocimum sanctum*, *Cassia fistula*, and *Azadirachta Indica*.

## Conclusions

Medicinal plants are sources of a large number of active principles of herbal and modern medicine. Indian people have a tremendous passion for medicinal plants and use them for a wide range of health related applications from a common cold to cancer and treatment of poisonous snake bites to a cure for genetic disorders like muscular dystrophy. India has one of the richest herbal medical cultures in the world that is of tremendous contemporary relevance ensuring health security to millions of people. However, continuous increase in environmental pollution is leading to built up of these pollutants including heavy metals in the plant parts which eventually enter the human food chain. Therefore, regular screening of raw material is must to check the levels of these pollutants in the plant parts and extracts before using them for human consumption. The present investigation clearly demonstrated the variation in heavy metal concentration depending upon the collection sites. Different countries have proposed different national limits for various heavy metals in raw herbal materials e.g the minimum permissible limits for arsenic, lead, cadmium, mercury and chromium are 5.0, 10.0, 0.3, 0.2 and 2.0 ppm respectively in Canada while the same are 2.0, 10.0, 1.0, 0.5 ppm respectively in China, and the limit has not yet been prescribed for chromium (WHO, 2007). However, the permissible limits have not been prescribed for many metals which are considered micronutrients. The developed AAS technique is a precise, specific and accurate method for estimation of heavy metal content in stem and leaf samples of these medicinal plants. Assessment of heavy metals in medicinal plants will pave the way for excluding extensively polluted environmental sites for collection of raw materials required for herbal drug preparation.
